# When Weight Loss Turns Dangerous: A Case Report of Tirzepatide-Induced Ketoacidosis in a Non-diabetic Woman

**DOI:** 10.7759/cureus.113368

**Published:** 2026-07-25

**Authors:** Humaid Sadiq, Zahra Amini, Mansoor M Husain, David O Alao, Thiagarajan Jaiganesh

**Affiliations:** 1 Department of Emergency Medicine, Tawam Hospital, Al Ain, ARE; 2 Department of Internal Medicine, Emergency Medicine Section, United Arab Emirates University, College of Medicine and Health Sciences, Al Ain, ARE

**Keywords:** emergency medicine, glp-1 receptor agonist, ketoacidosis, obesity, tirzepatide

## Abstract

Tirzepatide has emerged as an effective therapeutic option for the management of obesity and type 2 diabetes mellitus because of its substantial benefits in weight reduction and glycemic control. Gastrointestinal adverse effects are common, whereas serious metabolic complications such as ketoacidosis remain rare. We describe the case of a woman in her early 30s with class II obesity who developed persistent vomiting, epigastric pain, and fatigue six days after her first tirzepatide injection. Laboratory investigations revealed mild hypoglycemia and high-anion-gap metabolic acidosis with markedly elevated ketone levels. Following an initial treatment in the emergency department (ED), she was admitted to the intensive care unit (ICU). She was managed with intravenous fluids, electrolytes, dextrose, and insulin, and she made a good recovery within 48 hours. The case highlights the potential life-threatening complications of dual incretin receptor agonists and the need to increase clinician awareness.

## Introduction

Tirzepatide has emerged as an effective therapeutic option for the management of obesity and type 2 diabetes mellitus because of its substantial benefits in weight reduction and glycemic control [1,2]. As the first dual glucose-dependent insulinotropic polypeptide (GIP) and glucagon-like peptide-1 (GLP-1) receptor agonist, it represents a novel therapeutic approach that has demonstrated superior efficacy compared with several existing incretin-based therapies in clinical trials [1-3]. Its unique pharmacologic profile, which includes biased GLP-1 receptor signaling and GIP receptor potentiation, enhances insulin secretion, delays gastric emptying, and promotes appetite suppression [4]. Consequently, its clinical use has expanded rapidly following approval for treatment of obesity [2,3].

Gastrointestinal adverse effects, particularly nausea, vomiting, and reduced oral intake, are the most frequently reported complications of tirzepatide therapy and are generally mild and self-limiting [2,3]. However, emerging evidence suggests that these effects may, in rare circumstances, precipitate starvation-related or euglycemic ketoacidosis, even in individuals without diabetes [5,6]. Starvation ketoacidosis occurs when prolonged caloric and carbohydrate restriction leads to accelerated lipolysis and hepatic ketone production despite normal or only mildly elevated serum glucose concentrations [7]. Although only a handful of cases have been reported in the literature [5,6,8-10], delayed recognition may result in severe metabolic derangement requiring intensive care management.

We present the case of a woman in her early 30s with obesity who developed severe ketoacidosis six days after her first tirzepatide injection. This case highlights a rare but potentially life-threatening adverse effect of dual incretin receptor agonist therapy and emphasizes the importance of early recognition and prompt metabolic evaluation in patients presenting with persistent gastrointestinal symptoms following tirzepatide use.

## Case presentation

Clinical presentation

A woman in her early 30s with obesity presented to the emergency department with persistent vomiting that began six days after receiving her first 5 mg dose of tirzepatide for weight loss. She had no history of diabetes mellitus. Vomiting occurred three to four times daily and was associated with burning epigastric pain radiating to the chest, diffuse abdominal discomfort, and marked fatigue. She was unable to tolerate solid food but could ingest small amounts of water. She denied fever, chills, shortness of breath, cough, diarrhea, or dysuria, and had not passed stool for four days. She denied use of sodium-glucose transport protein 2 (SGLT2) inhibitors, corticosteroids, alcohol, or other medications associated with ketoacidosis.

Clinical assessment

On examination, she was alert and oriented. Her vital signs were as follows: blood pressure 116/77 mmHg, heart rate 98 beats/min, respiratory rate 20 breaths/min, and temperature 36.7°C. Abdominal examination revealed epigastric tenderness without guarding or other peritoneal signs.

Diagnostic evaluation

**Figure 1:**
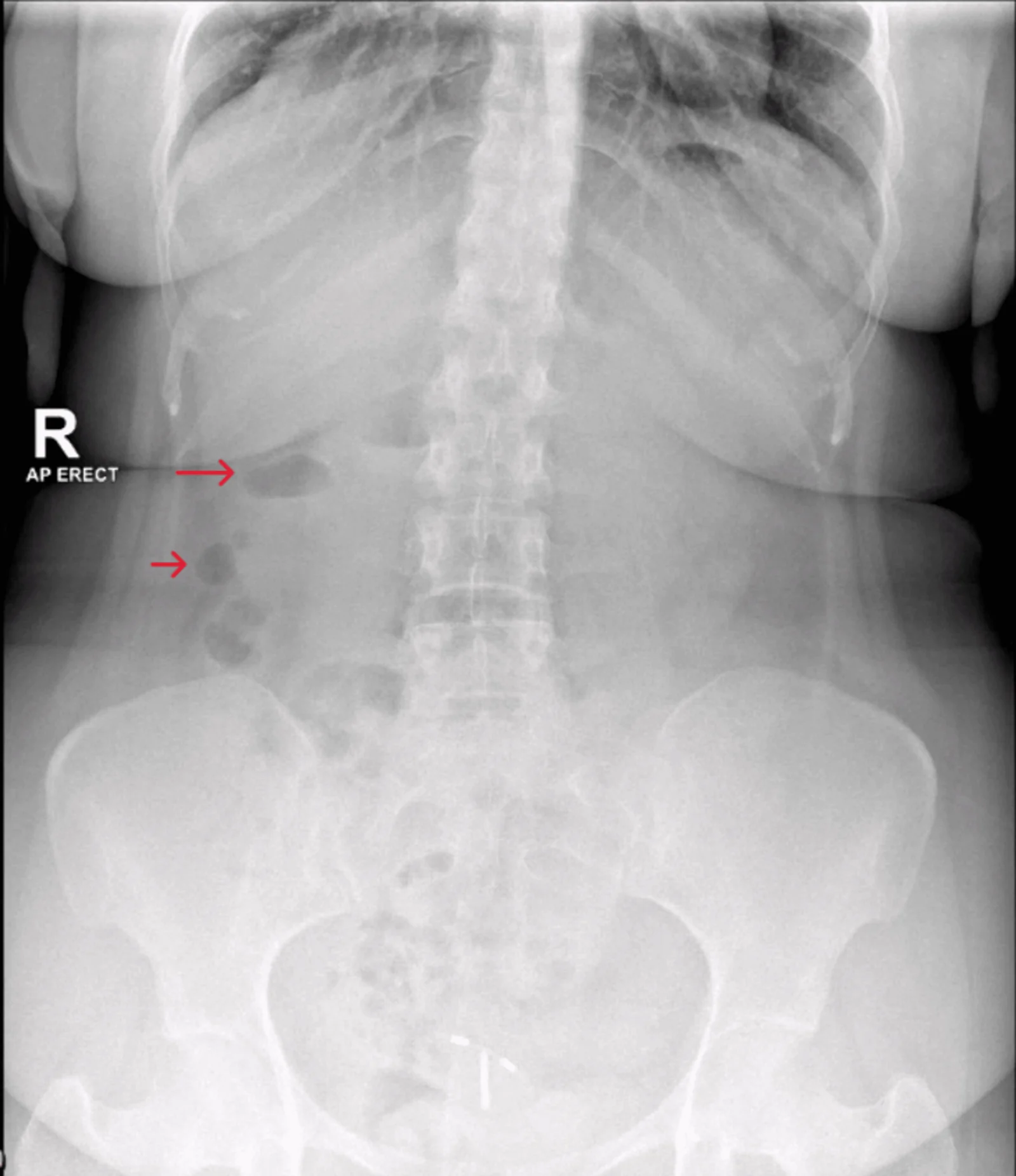
Chest and abdominal radiograph showing no evidence of bowel obstruction or pneumoperitoneum. Chest and abdominal radiograph demonstrating normal cardiac silhouette and clear lung fields. The abdominal gas pattern is unremarkable (as indicated by the red arrows) with no air-fluid levels or subdiaphragmatic free air. No radiopaque pathological shadows are seen. An IUCD is incidentally noted in situ. IUCD: intrauterine contraceptive device.

**Figure 2:**
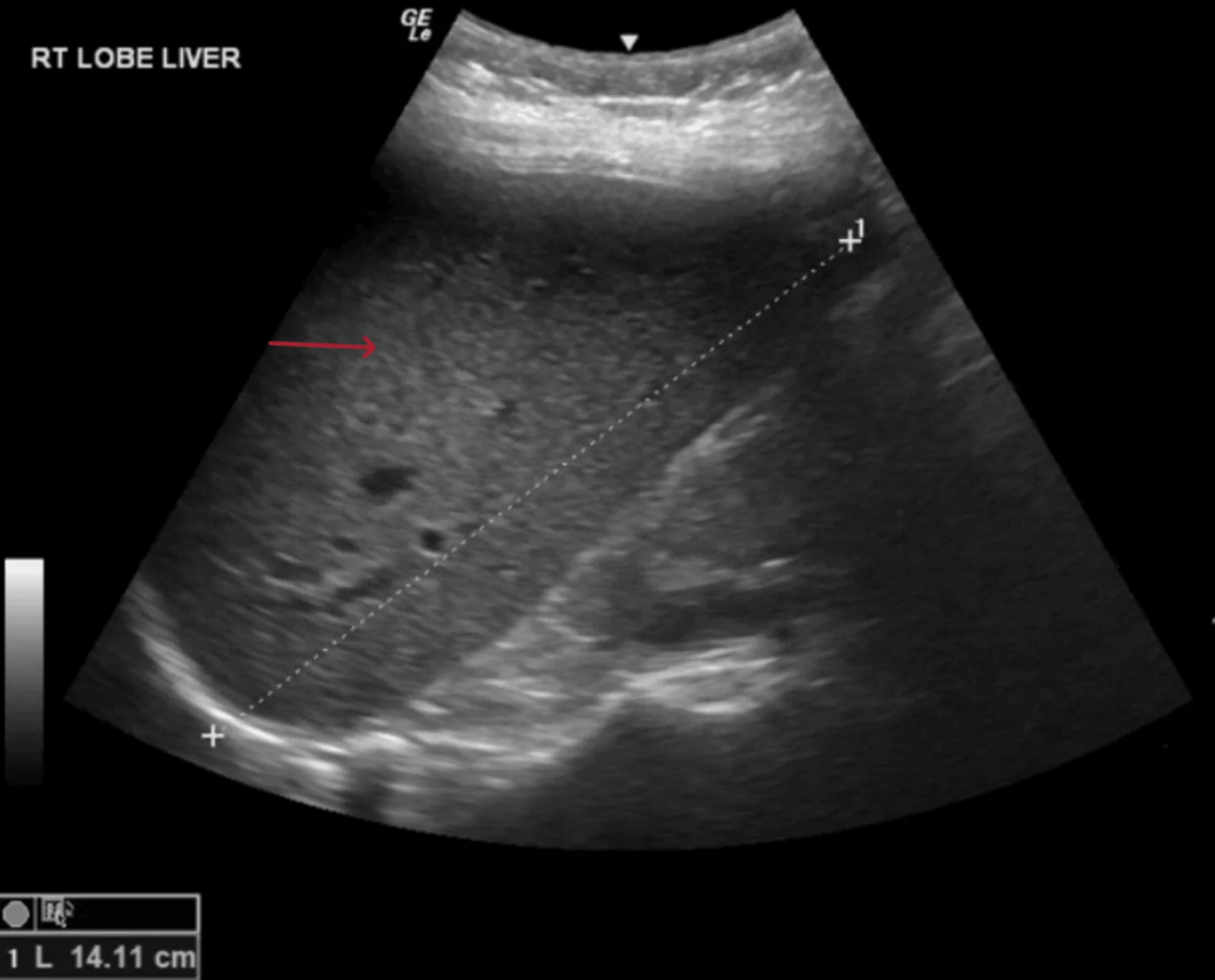
Hepatobiliary ultrasound demonstrating normal liver parenchyma (as indicated by the red arrow) and no biliary pathology. Ultrasound examination of the hepatobiliary system demonstrating normal liver size, smooth contour, and homogeneous echotexture without focal lesions. The portal vein is normal in caliber. The gallbladder shows no wall thickening, pericholecystic fluid, or intraluminal sludge. The common bile duct and intrahepatic biliary ducts are within normal limits. The pancreas is partially obscured by bowel gas but the visualized portions are unremarkable. No ascites or lymphadenopathy is identified.

Initial venous blood gas analysis demonstrated a pH of 7.16, bicarbonate of 11 mmol/L, base excess of −16.1, an anion gap of 19, and glucose of 3.5 mmol/L, consistent with high-anion-gap metabolic acidosis with mild hypoglycemia (Table 1). Serum electrolytes showed mild hyponatremia (133 mmol/L) with normal potassium (3.7 mmol/L). Serum beta-hydroxybutyrate was markedly elevated at 6.4 mmol/L (reference range <0.5 mmol/L). Renal function tests, pancreatic enzymes, troponin-I, and C-reactive protein (Table 2), as well as complete blood count and liver function tests, were unremarkable. Chest and abdominal radiography and hepatobiliary ultrasonography demonstrated no acute intra-abdominal pathology (Figures 1, 2).

**Table 1:** Initial venous blood gas investigation on emergency department presentation.

Test	Result	Reference range	Unit
pH	7.16	7.35-7.45	
pCO_2_	31.6	35.0-45.0	mmHg
pO_2_	25.4	25.0-40.0	mmHg
HCO_3_	11	22-26	mmol/L
Potassium (K)	4.1	3.4-5.1	mmol/L
Chloride (Cl)	110	98-107	mmol/L
Sodium (Na)	140	136-145	mmol/L
Base excess (BE)	-16.1	-2.0 to 2.0	mmol/L
Total hemoglobin (THB)	168	120-170	g/L
Carboxyhemoglobin (COHB)	1.2	0.0-2.0	%
Glucose (Glu)	3.5	3.9-6.0	mmol/L
Lactate (Lac)	1.1	0.5-2.2	mmol/L

**Table 2:** Other laboratory investigations during emergency department stay.

Test	Result	Reference range	Unit
Sodium (Na)	133	136-145	mmol/L
Potassium (K)	3.7	3.5-5.1	mmol/L
Chloride (Cl)	107	98-107	mmol/L
Beta ketones	6.4	0.1-0.6	mmol/L
Creatinine	53.5	44.2-88.4	micromol/L
Urea	2.00	2.5-6.70	mmol/L
Estimated glomerular filtration rate (eGFR)	122	(≥60)	mL/min
Lipase	106	13-60	IU/L
Troponin-I	<10.00	(≤15.60)	ng/L
C-reactive protein	22.0	(≤5.0)	mg/L

Emergency department management

The patient received 1 L of intravenous normal saline, intravenous acetaminophen 1 g, intravenous metoclopramide 10 mg, and intravenous pantoprazole 40 mg. Given persistent metabolic acidosis with elevated ketones, she was started on 10% dextrose infusion (250 mL) in addition to ongoing fluid resuscitation. Despite initial resuscitation, metabolic acidosis persisted, and ketone levels remained elevated (Table 3), prompting admission to the intensive care unit (ICU) for close monitoring and ongoing management.

**Table 3:** Subsequent laboratory investigations during emergency department stay.

Test	Result	Reference range	Unit
pH	7.12	7.35-7.45	
pCO_2_	33.9	35.0-45.0	mmHg
pO_2_	23.2	25.0-40.0	mmHg
HCO_3_	11	22-26	mmol/L
Base excess (BE)	-17.2	-2.0 to 2.0	mmol/L
Potassium (K)	3.6	3.4-5.1	mmol/L
Chloride (Cl)	113	98-107	mmol/L
Sodium (Na)	140	136-145	mmol/L
Beta ketones	6.2	≤5.0	mmol/L
Lactate (Lac)	1.1	0.5-2.2	mmol/L

Intensive care course

In the ICU, she received intravenous fluids, electrolyte replacement, dextrose, and insulin as indicated. Potassium replacement was administered based on serial electrolyte monitoring during her ICU stay. Her nausea and vomiting resolved, oral intake gradually improved, and serial laboratory investigations demonstrated progressive resolution of the metabolic derangements.

Outcome and follow-up

Her metabolic derangements resolved completely, and she was discharged home after three days of hospitalization. Follow-up investigations demonstrated a normal HbA1c (4.9%), and hepatobiliary imaging showed no abnormalities. She remained asymptomatic during follow-up without recurrence of symptoms.

## Discussion

Tirzepatide is a dual GIP and GLP-1 receptor agonist that has transformed the management of type 2 diabetes mellitus and obesity [4]. Through enhancement of insulin secretion, delayed gastric emptying, and appetite suppression, it produces significant improvements in glycemic control and weight reduction [1,2]. Large clinical trials, including SURPASS and SURMOUNT, have demonstrated its superior efficacy compared with existing therapeutic options [1-3].

Despite these benefits, tirzepatide is associated with gastrointestinal adverse effects such as nausea, vomiting, and reduced appetite [1-3], which may, in rare cases, precipitate metabolic complications including ketoacidosis [5-10]. In our patient, high-anion-gap metabolic acidosis with marked ketonemia developed six days after initiation of tirzepatide, with no alternative precipitating cause identified.

The underlying mechanism is likely multifactorial. Gastrointestinal intolerance leading to reduced oral intake, vomiting, and prolonged caloric deprivation can result in glycogen depletion and increased lipolysis, promoting ketogenesis [4,7]. This may culminate in starvation-related ketoacidosis, which can occur despite normal or low serum glucose levels.

Previously reported cases of tirzepatide-associated ketoacidosis in non-diabetic individuals describe a similar pattern of severe gastrointestinal symptoms followed by metabolic decompensation after initiation of therapy [5,6,8-10]. In line with these reports, our patient developed significant ketoacidosis despite the absence of diabetes, supporting a possible association between tirzepatide-induced gastrointestinal intolerance and starvation-related ketoacidosis. However, causality cannot be established from isolated case reports. Notably, in our case, severe metabolic derangement occurred within six days of the first dose, highlighting that this complication may occur early in therapy.

Although available reports remain limited, persistent gastrointestinal symptoms with reduced oral intake appear to be the predominant preceding feature [5-8]. Patients using tirzepatide for weight loss or those with additional risk factors such as dehydration, low carbohydrate intake, or intercurrent illness may be particularly susceptible.

The main limitation of this report is that, as a single-case report, a definitive causal relationship between tirzepatide use and ketoacidosis cannot be established. Nevertheless, the close temporal association, exclusion of alternative causes, and consistency with previously published cases support a possible association warranting further investigation.

Patients should be counselled regarding warning symptoms including persistent vomiting, abdominal pain, fatigue, and inability to maintain oral intake. In symptomatic individuals, early evaluation with serum ketones, venous blood gas analysis, and electrolytes should be considered even in the absence of diabetes or marked hyperglycemia [7]. Alternative causes of high-anion-gap metabolic acidosis, including diabetic ketoacidosis, alcoholic ketoacidosis, toxic alcohol ingestion, lactic acidosis, sepsis, and pancreatitis, should also be considered and excluded based on the clinical presentation and laboratory findings.

From an emergency medicine perspective, persistent vomiting following tirzepatide initiation should prompt consideration of ketoacidosis in both diabetic and non-diabetic patients. Early diagnostic evaluation with venous blood gas, serum ketones, and electrolytes is essential when gastrointestinal symptoms are accompanied by unexplained fatigue or reduced oral intake. Management involves correction of volume depletion, electrolyte abnormalities, and suppression of ketogenesis using intravenous fluids, dextrose, and insulin when indicated [7]. In severe cases, early intensive care involvement facilitates close metabolic monitoring and guided therapy.

## Conclusions

Tirzepatide, while effective for weight management and glycemic control, may rarely be associated with ketoacidosis in non-diabetic patients, particularly in the setting of persistent vomiting or reduced oral intake. Prompt recognition, laboratory assessment, and supportive management are critical to achieving favorable outcomes. Clinicians should remain vigilant for this uncommon but potentially life-threatening adverse event, particularly as the use of tirzepatide expands. Reporting additional cases will improve awareness, inform safe prescribing practices, and help clarify the incidence, underlying mechanisms, and risk factors for ketoacidosis in non-diabetic populations.
